# Fertility intentions and outcomes in Indonesia: Evolutionary perspectives on sexual conflict

**DOI:** 10.1017/ehs.2021.27

**Published:** 2021-05-06

**Authors:** Kristin Snopkowski, James Joseph Nelson

**Affiliations:** Department of Anthropology, 1910 University Drive, Boise State University, Boise, ID 83725, USA

**Keywords:** desired fertility, ideal family size, sexual conflict, Indonesia

## Abstract

Differential fertility preferences for men and women may provide insights into human sexual conflict. We explore whether pairbonded couples have different preferences for future offspring, which socioecological factors are associated with these preferences, and who achieves their desired fertility over time. We utilise the Indonesia Family Life Survey (IFLS), a longitudinal survey which collected data from 1993 to 2015, to compare desired future fertility for 9655 couples and follow couples who had divergent preferences. The majority of couples (64.8%) want the same number of future offspring. In 20.7% of couples, husbands want more future offspring than their wives, while the reverse occurs in 14.5% of couples. Living in villages with the husband's or the wife's parent(s) is associated with having divergent preferences for future offspring, where there is a higher likelihood that women prefer more offspring than their husbands. When examining fertility outcomes, women, particularly those who marry at older ages, are more likely to achieve their desired preference. Contrary to previous research, we do not find that living near one's natal kin or having increased autonomy increases an individual's likelihood of achieving desired fertility outcomes.

## Graphical abstract

**Figure:**
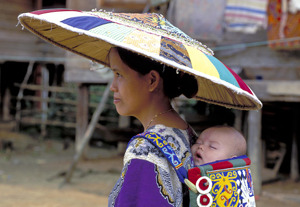

**Social media summary:** Who wants more children and who achieves their preference? A longitudinal study of Indonesian couples finds out.

Studies that examine fertility intentions find that when differences occur between men and women, it is typically women who want fewer children than men, particularly in Sub-Saharan Africa (Bankole & Singh, 1998; Borgerhoff Mulder, 2009; Nii-Amoo Dodoo, 1993; Ratcliffe, Hill, & Walraven, 2000). In contrast, when examining couples, the majority agree on whether to have additional children – particularly when examining contexts outside of Sub-Saharan Africa (Mason & Smith, 2000; Mason & Taj, 1987; Moya, Snopkowski, & Sear, 2016). It is simultaneously possible that men want more children than women across multiple partners, but that within a particular pairbond, men and women have similar preferences. Differences in desired fertility may represent sexual conflict, where one partner wants to continue having offspring and the other would prefer to delay or cease reproduction.

Sexual conflict is defined as ‘a conflict between the evolutionary interests of individuals of the two sexes’ (Arnqvist & Rowe, 2005; Parker, 1979) or more precisely ‘sexually antagonistic selection on shared traits’ (Rowe & Day, 2006). This occurs when selection favours sex-specific reproductive strategies that increase the reproductive success of one sex while simultaneously reducing (or constraining) fitness of the other sex (Aloise King, Banks, & Brooks, 2013; Stumpf, Martinez-Mota, Milich, Righini, & Shattuck, 2011). Sexual conflict is frequently displayed and documented in promiscuous species where males exploit female optima in an attempt to increase their own fitness (Barkow & Burley, 1980) through mechanisms such as limiting female mate choice (Chapman, 2018; Clutton-Brock & Parker, 1995; Smuts & Smuts, 1993) or increasing investment/production of offspring for a given reproductive event above the female's optimal level (Chapman, 2001; Haig, 2010). Females exhibit their own adaptations to counter male strategies to co-opt their fitness optima (Chapman, 2018; Mulder & Rauch, 2009). Sexual conflict is reduced when the relative proportion of a female's progeny is fathered by a single male (Barkow & Burley, 1980). Sexual conflict is eliminated when individuals reproduce with one, and only one, mate for their entire lives (Holland & Rice, 1999). In this case, each individual's fitness is directly tied to their partner – so maximising reproductive success of one individual simultaneously maximises it for their partner. Humans engage in long-term pairbonding but do not mate exclusively with one partner for life. This suggests that sexual conflict should be lower in humans than in other species where pairbonding is rare, but that it is not absent.

## Sexual conflict over fertility preferences

Differential fertility preferences, defined as the desired or ideal number of offspring for men and women, may represent sexual conflict *if* this represents different optimal fertility rates across the sexes. Differences in fertility preferences do not, by themselves, indicate sexual conflict as maximisation of non-fitness currencies may also explain these differences, see below. Sexual conflict is frequently explained as the result of asymmetries in the minimum investment required for reproduction for males and females (Aloise King et al., 2013), although a variety of factors need to be considered (Kokko & Jennions, 2008). Human females bear higher reproductive costs for each reproductive event than human males. Pregnant and lactating women require between 200 and 630 additional kilocalories per day (Butte & King, 2005; Jasienska, 2020). Males, in contrast, have substantially lower minimal energetic demands for reproduction, only that to produce sperm (but note that substantial energy may be needed to acquire a mate; Moya et al., 2016). Investment in human offspring, though, requires extensive investment beyond that needed to birth and wean an offspring. While mothers in the majority of non-human primate species are the exclusive caretaker for infant offspring, humans are unique in that many individuals, in addition to the mother, provide care for offspring (Helfrecht, Roulette, Lane, Sintayehu, & Meehan, 2020; Hrdy, 2005, 2009; Mace & Sear, 2005; Sear & Mace, 2008). Human males may have minimal obligate investment in offspring, but their actual investment is typically much larger. Human paternal investment varies both within and between societies, with many men providing substantial direct or indirect care for offspring, and others providing little to none (Geary, 2000; Hewlett, 1991). It has been hypothesised that differential cost of reproduction corresponds to disparate preferences for ideal number of children over one's lifetime – children conceived across multiple mates (Mulder & Rauch, 2009; Penn & Smith, 2007), but it is not necessarily the case that within a reproducing couple differential costs explain differences in desired family size (Moya et al., 2016). For instance, if women experience higher mortality costs following childbirth than men, some might expect that women would want to have fewer children than men because of this mortality risk – but men should incorporate the risk of their partner's mortality when attempting to optimise reproduction within that long-term pairbond. If a man tries to persuade or exploit a woman to increase her reproduction to a level that is maladaptive, he will be hurting his reproductive success within that pairbond as well. Of course, this assumes that people are optimising their fertility, that ‘costs’ are fitness-related and that men have consistent paternity certainty over time within a given pairbond, none of which are necessarily true (for a more detailed discussion of the factors that influence optimal male and female reproductive rates, see Moya et al., 2016).

Hypothesis 1: *Men have higher ideal family sizes than women, but within reproducing couples, desire similar numbers of future offspring.*

Modelling work suggests that, in humans, where long-term pairbonds are the norm and men and women's reproductive success are closely tied, exploiting female reproduction to maladaptive levels hurts both male and female fitness, except under very particular circumstances (Barkow & Burley, 1980; Moya et al., 2016). While the conditions where men exploit female fertility to maladaptive levels are expected to be limited, we may expect men to prefer women to limit their reproduction more than is optimal for women. For instance, when paternity certainty decreases with time, men should want women to reduce their future reproduction more than women do, allocating investments in current offspring that he knows are his (Barkow & Burley, 1980; Moya et al., 2016). Based on these models, we predict that spousal age difference, where men are significantly older than their mates, may be associated with differential fertility preferences, where men have lower desired fertility than their much younger wives, since they experience decreasing paternity certainty given mortality risks (Barkow & Burley, 1980).

Hypothesis 2a: *Men have lower fertility intentions than women when husbands are significantly older than their wives.*

## Fertility preferences depend on socioecological context

It is also possible that differential fertility preferences are not the result of simple sex differences, but the result of one's socioecology. Theoretical models of kin conflict suggest that female-biased dispersal (which is associated with patrilocal postmarital residence) may set up a reproductive conflict where daughters-in-law have an advantage over older mothers-in-law, since they have more to lose by forgoing reproduction owing to relatedness asymmetries (Cant & Johnstone, 2008). This model provides an explanation for the higher rate of reproduction among women living patrilocally after marriage (Colleran, 2013; Sear & Coall, 2011; Sear, Moya, & Mathew, 2013; Snopkowski, Moya, & Sear, 2013; Snopkowski & Sear, 2013). In contrast, when adult children live in communities with their own parents, reproductive conflict should favour the older generation, since parents have more to lose if they forgo reproduction. From the adult child's perspective, they are equally related to a full sibling as a biological offspring and therefore will be more willing to forgo reproduction (Moya & Sear, 2014). If young women living in patrilocal contexts can win reproductive conflicts with their mothers-in-law, we might predict that women living patrilocally have higher desired fertility than their husbands (who experience the same inclusive fitness benefit if they have a biological offspring or full sibling).

Hypothesis 2b: *Women living patrilocally will have higher desired fertility than their husbands.*

We can also frame these preferences as the outcome of kin cooperation. Individuals who live near their own kin may allocate investment towards their indirect fitness (individuals other than biological offspring, such as nieces, nephews, siblings, etc.) instead of directing investment exclusively towards biological offspring. Mathematical modelling has shown that women may delay first births when they have full siblings to invest in as compared with when they have half siblings (Moya & Sear, 2014). Women may prefer to have fewer offspring when they live near their kin, which is more likely when postmarital residence is matrilocal (Johnstone & Cant, 2010). In contrast, if men are living near their wife's kin, they may prefer to provide investments in their biological offspring over non-related individuals.

Hypothesis 2c: *Women living matrilocally will have lower desired fertility than their husbands.*

Conversely, people may prefer to increase their reproduction when they live near kin if those kin are likely to act as alloparents or provide support to the reproducing couple (Turke, 1989), or if kin provide pronatal messages (Newson et al., 2007). Evidence has shown that women are more likely to have additional offspring when kin provide childcare or other (financial or emotional) support (Kaptijn, Thomese, van Tilburg, & Liefbroer, 2010; Mathews & Sear, 2013; Schaffnit & Sear, 2017; Snopkowski & Sear, 2016; Waynforth, 2012). In this situation, we may expect that women have higher desired fertility in matrilocal contexts or when women live near their kin. The role of patrilocal contexts is less clear. If women perceive their affinal kin as providing alloparental (or other) support, then women may have similar preferences in matrilocal and patrilocal contexts (both more than in contexts where women live far from kin).

Hypothesis 2d: *Women living near kin will have higher desired fertility than their husbands*.

It is hard to predict how men's fertility preferences may respond to cues of alloparental help. They may similarly find that kin support increases their desired fertility, or be less sensitive to cues of alloparental care, particularly in contexts where women take the primary role for direct caregiving. If the preferences of both men and women move in concert, it will be undetectable in an analysis of differential fertility preferences.

## Fertility preferences as the result of optimisation of non-fitness currencies

Finally, it is possible that differential fertility preferences are the result of people optimising non-fitness currencies (Moya et al., 2016). Women may prefer to pursue activities beyond motherhood (McAllister, Gurven, Kaplan, & Stieglitz, 2012; Newson, Postmes, Lea, & Webley, 2005) and motherhood may be more incompatible with other activities than fatherhood (Tiefenthaler, 1997). As an example, in some contexts women may experience higher opportunity costs of reproduction than men related to employment (Browning, 1992). Career-aspiring women may prefer smaller family sizes than men, and factors, like education, which provide greater opportunity for employment may associate with fertility preferences.

Hypothesis 2e: *Women with more education will have lower desired fertility than their husbands owing to increased opportunity costs.*

Why might people optimise non-fitness currencies? There are two possibilities: people may simply pursue activities that are maladaptive given the novel environments we currently live in or people may pursue activities that were correlated with reproductive success in our evolutionary past, but are no longer correlated (e.g. status-seeking activities). If women seek to achieve status, then in contexts where production of offspring is the only route to status – particularly among affinal kin – they may prefer to have more offspring (Armitage, 1993). In contexts where wealth, education or career success leads to status, women may seek these attributes at the expense of reproduction (Shenk, Kaplan, & Hooper, 2016).

To test the above hypotheses, we will examine fertility intentions among Indonesian couples. Specifically, we ask: (1) Is there a difference in (a) desired future reproduction and (b) ideal family size among married men and women in Indonesia (testing Hypothesis 1); and (2) if so, which variables are associated with differential desired fertility (testing Hypotheses 2a–e)?

## Resolution of fertility intentions

Stated fertility intentions are predictive of future fertility, even though they may be imperfect (Schoen, Astone, Kim, Nathanson, & Fields, 1999; Yeatman, Trinitapoli, & Garver, 2020). We examine how differences in fertility intentions among men and women may be resolved over time. We expect that fertility outcomes are the result of negotiations over fertility preferences by wives and husbands, with possible influences of their kin (Borgerhoff Mulder, 2009). Our third research question asks: how are fertility intention differences (when they exist) resolved? Under which socioecological contexts do individuals achieve their preferred number of offspring? Below we discuss hypotheses related to these questions.

Evidence suggests that fertility is reduced when women have more autonomy (Jejeebhoy, 1991; Upadhyay et al., 2014), with the assumption that women desire lower fertility and increased autonomy allows for its achievement.

Hypothesis 3a: *When women have more autonomy, they are more likely to achieve their desired fertility.*

Women's autonomy has been associated with smaller spousal age gap, that is, couples with older husbands and substantially younger wives are associated with reduced female autonomy (Barbieri & Hertrich, 2005; Carmichael, 2011; cf. Lawson et al., 2020). This leads to Hypothesis 3b.

Hypothesis 3b: *When the spousal age gap is larger, men will be more likely to achieve their desired fertility.*

Women's autonomy may also be associated with their access to natal kin. A frequent empirical finding in the evolutionary literature is that couples have more offspring when living patrilocally (Colleran, 2013; Sear & Coall, 2011; Sear et al., 2013; Snopkowski et al., 2013; Snopkowski & Sear, 2013). This could represent sexual conflict over family size that is resolved in favour of the husband's preference – with the assumption that men and their kin prefer women to have more offspring than they want (Leonetti, Nath, & Hemam, 2007) and that in these contexts men are able to achieve their preference. Empirical evidence from Karachi, Pakistan has shown that mothers-in-law have higher stated fertility preferences than their daughters-in-law (Kadir, Fikree, Khan, & Sajan, 2003) and other research has found that women are less likely to use contraceptives when their husbands or mothers-in-law oppose their use (Blackstone, Nwaozuru, & Iwelunmor, 2017; Onwuzurike & Uzochukwu, 2001; Qutub, 1995). Women may be able to achieve their desired fertility preference when they live near their natal kin, while men achieve their desired preference when they live near their kin.

Hypothesis 3c: *Women living matrilocally (or near their kin) will be more likely to achieve their desired fertility, while women living patrilocally (or away from their natal kin) will be less likely to achieve their desired fertility.*

Different fertility preferences may also be resolved in the direction of the more normative behaviour. Previous research has shown that couples are more likely to refrain from having additional children in low-fertility contexts if couples disagree about wanting another child, while couples in high-fertility contexts are more likely to have additional children if couples disagree about wanting another child (DaVanzo, Peterson, & Jones, 2003; Voas, 2003). This leads to Hypothesis 3d.

Hypothesis 3d: *In low fertility contexts, couples will be less likely to have more children if they disagree over whether to have additional offspring.*

## Prior research

Several prior studies have examined family size preferences, sexual conflict and reproductive outcomes. A study of the Mpimbwe, a horticultural society in Western Tanzania, found that men desire more children than women, but having a consistent husband does *not* result in higher overall fertility or number of surviving offspring for women approximately 10 years later (Borgerhoff Mulder, 2009). A survey of parents from Yokohama City, Japan found that, while women reported higher costs of childcare, there was no conflict over ideal family size and both mothers and fathers reported that they had equal power to make reproductive decisions (Morita, Ohtsuki, & Hiraiwa-Hasegawa, 2016). A study of the Tsimane, a lowland South Amerindian forager–horticulturalist group in Bolivia, found that women tend to have more children than their stated ideal family size, but that couples where the husband wants additional children and the wife does not are *not* more likely to have additional children than couples who agree (McAllister et al., 2012). A study of the Yoruba of Nigeria found that the fertility desires of both partners are important predictors of overall fertility and that when spouses disagree, intermediate fertility tends to be the result (Bankole, 1995). Overall, previous studies suggest relatively tepid evidence for husbands achieving their fertility preferences at the expense of their wives.

## Methods

We utilise data from the Indonesia Family Life Survey (IFLS) to examine fertility preferences and resolution among Indonesian couples. The IFLS is a longitudinal survey that collects individual- and community-level information on fertility, education, employment, migration and health (Frankenberg & Karoly, 1995; Frankenberg & Thomas, 2000; Strauss et al., 2004; Strauss, Witoelar, & Sikoki, 2016; Strauss, Witoelar, Sikoki, & Wattie, 2009). Surveys were conducted across five waves: collected in 1993/1994, 1997/1998, 2000, 2007/2008 and 2014/2015. IFLS data, which is managed by the RAND Corporation, is publicly available, but cannot be distributed by users and requires registration at: https://www.rand.org/well-being/social-and-behavioral-policy/data/FLS/IFLS/access.html. The survey represents an area that includes 83% of Indonesia (13 provinces found on the islands of Java, Sumatra, Bali, West Nusa Tenggara, Kalimantan and Sulawesi). Small provinces and provinces that were politically unstable at the time of the first interview were not sampled. A total of 7224 households were surveyed in 1993/1994 (Frankenberg & Thomas, 2000). In each subsequent wave, the original household members who completed a detailed interview in 1993/1994 were re-interviewed and household members who did not complete a detailed interview have been systematically added throughout the waves (Frankenberg & Thomas, 2000; Strauss et al., 2004, 2009). When households split, both households are interviewed in subsequent waves. A total of ~15,900 households were interviewed in 2014/2015. Analyses were conducted in STATA v. 13. The STATA do file is available at: https://osf.io/ygpmh/?view_only=872ec8eeb9a142bdb81cc8d7b9293c3b.

### Cultural context

Indonesia is the fourth most populous country in the world and is extremely diverse, with over 300 ethnic groups utilising varying kinship systems (Rammohan & Johar, 2009). For example, the Balinese people, who practise Hinduism, tend to be patrilocal (Jensen & Suryani, 1992), the Minangkabau people of West Sumatra are matrilineal and matrilocal (Krier, 2000), and other ethnic groups, like the Sumbawanese and the Bima-Dompu, are multilocal (Lebar, 1972; Murdock, 1967; Snopkowski, Moya, & Sear, 2014). Over the past 50 years, Indonesia has experienced a dramatic reduction in fertility. The average number of children born per woman (total fertility rate) has fallen from 5.7 in 1960 and 2.8 in 1993 to 2.4 in 2015 (The World Bank, 2019). During this time, there was economic growth, particularly during the 1970s and 1980s (van der Eng, 1992), and structural changes occurred in agriculture and manufacturing (Soesastro & Chatib, 2005). A major economic downturn occurred in 1997–1998, known as the Asian Financial Crisis (Elias & Noone, 2011). Indonesia experienced rapid urbanisation with 17.1% of people living in urban areas in 1960 transitioning to 52.6% in 2009 (Lewis, 2013). Indonesia is the largest Muslim majority country in the world, with 86% of participants in 1993/1994 identifying as Muslim. Arranged marriage was once common (over 50% of rural women born before 1953) and was associated with high divorce rates and self-choice remarriage, but has become less frequent with time (Heaton, Cammack, & Young, 2001; Malhotra, 1991). In the decades preceding the IFLS, Indonesia experienced a dramatic increase in primary school attendance and developed a state-sponsored family planning programme (Molyneaux & Gertler, 2000). Indonesia has nearly universal marriage (97% of women in the IFLS are married by age 30). Evidence from the Indonesia Young Adult Reproductive Health Survey suggests low rates of non-marital fertility (only a few pregnancies outside of marriage were reported out of over 8000 women interviewed) as pregnancy among unmarried women is socially unacceptable (BPS Statistics Indonesia and Macro International, 2008).

### Samples

We utilise two samples throughout. The first is the sample of couples (married once, monogamously, wife is 35 years old or younger) who report their desired number of future offspring (*n* = 9655) to examine questions related to desired fertility. By limiting our sample to couples in their first marriage, we assume that both the wife and husband have the same number of current children (given low rates of non-marital fertility), allowing us to compare how many additional children they desire. We restrict the age of women to allow time for future fertility. Including women up to 40 or 45 primarily increases the number of couples who do not desire additional children. The second sample includes couples (married once, monogamously) who disagree on the number of future offspring desired. There were 3536 couples who had different preferences for future offspring. Of these, 1949 couples remained married, reported birth information and were followed for a minimum of 7 years. This can be further divided into 170 couples who had a number of children that fell between the wife's and husband's preferences and 1779 couples where one person ‘achieved’ their desired fertility.

### Data analysis

To determine whether there is a difference in desired future reproduction among married men and women, we examine couples to compare their answers to ‘do you personally wish to have another child (besides the children you already have)?’ and ‘how many (more) children do you wish to have?’ Starting in 1997/1998, participants could answer with ‘Up to God’. For these analyses, we include couples the first time they are asked, where both answered with a numerical value. We conduct a one-sample test of proportions to see if couples are evenly split (50%/50%) in which partner (wife or husband) prefers more offspring.

While ideal family size may be a less reliable indicator of future fertility, we can also examine whether there are differences between men and women. In this case, we do not need to limit the sample to those who are currently married, although the question was only asked of those who were *ever married*. Participants are included the first time they answer the question, regardless of their number of marriages or polygyny status. A potential response to the question, ‘if you could choose exactly the number of children to have in your whole life, how many would that be’ is ‘up to God’. This makes analysis more challenging, but we can examine how this changes for men and women across time, to determine whether there are differential preferences for ideal fertility. We also conduct a *t*-test to compare the ideal family size across men and women for those who provide numeric answers.

We conduct a multinomial logistic regression model to determine which attributes correspond to having the same preference for future offspring compared with different preferences – either the husband preferring more future children or the wife preferring more future children. We examined the following predictors: age difference between partners (Hypothesis 2a), postmarital residence (Hypotheses 2b–d), proximity to kin (Hypothesis 2d) and wife's education (Hypothesis 2e).

Postmarital residence is defined as neolocal if the couple did not live with either the wife's or husband's parents after marriage, matrilocal if they lived with the wife's mother or father after marriage and patrilocal if they lived with the husband's mother or father after marriage. Proximity to kin is a categorical variable indicative of the presence of kin at the time of interview (when the couple reports their preference for future offspring). Given that postmarital residence may be a proxy for kin availability, we use both measures. Our hypotheses are only explicitly about kin availability in Hypothesis 2d, but we believe that hypotheses 2b and 2c could also be framed as hypotheses about kin availability (or lack thereof). The categories of kin availability indicate whether at least one of the wife's parents or husband's parents live in the same village (co-residence is also included). The options include: neither set of parent(s) are living in the same village, the wife's parent(s) live in the same village, the husband's parent(s) live in the same village or both the husband's and wife's parent(s) live in the same village. Parent's location was not collected in 1997/1998, so we run analyses both with and without the kin availability measure. Wife's education is defined as a categorical variable indicating the highest level of education achieved, including none, grade school, junior high, secondary school, vocational secondary school and postsecondary education.

We include a variety of control variables in our multinomial logistic regression model that may influence desired fertility (DaVanzo et al., 2003). We control for household wealth. The household wealth variable was constructed as a factor of the number of rooms in the house, floor type, toilet type, electricity status, type of outer wall, whether the house has a telephone (1993/1994) or a television (1997/1998, 2000, 2007/2008, 2014/2015) and monthly household rent (or amount family would have to pay if renting their home; in 2014/2015). This variable has a mean of approximately 0 and a standard deviation of 1 in each wave. Larger values are indicative of wealthier households. We also include the difference between husband and wife's education level as husband's education may be important in addition to wife's education, but we cannot include both variables in the model given multicollinearity. Positive values of this variable indicate that the husband has more education than the wife. Wife's age at marriage, arranged marriage status, number of currently living children and wife's age at interview are also included as possible control variables given that they may influence how many future children couples want. Finally, religion, region, urban vs. rural residence and wave of data collection are also controlled for given fertility differences across these factors.

To examine who achieves their desired fertility, we use the sample of couples (married once, monogamously) who had divergent preferences for future offspring. We monitor them for as long as possible by identifying the most recent wave with data on fertility where they remain married (to each other, monogamously), but requiring a minimum duration of 7 years for follow-up to determine who was able to achieve their desired fertility preference (we also examine a cut-off of at least 10 years as a sensitivity analysis). In every wave of data collection, women respond to questions about their number of live births since the previous wave. We include all live births as an indication of the couple's fertility during the follow-up period. We code the couple by who ‘achieved’ their desired fertility. As an example, if a couple had divergent fertility preferences, where the wife wanted two children, but her husband only wanted one, we code the husband as achieving his fertility preference if the couple has zero or one child after the maximum duration of follow-up. We code the wife as achieving her fertility preference if the couple has two or more children over the same time period. We analyse this using a logistic regression model (the event indicates that the wife achieved her desired fertility) and include the same predictors as listed above: spousal age difference (Hypothesis 3b) and postmarital residence/kin availability (Hypothesis 3c), and add wife's autonomy (Hypothesis 3a).

Wife's autonomy is the average of her autonomy in each wave across the range of years that she is surveyed. Women's autonomy is measured by responses to the following questions:

*In your household, who makes decisions about: (a) your child's health, (b) large expensive purchases for the household, (c) whether you work, (d) whether you (and your spouse) use contraception and (e) your time spent socialising*.

Women report who makes these decisions and can report multiple people. Autonomy is measured as the average of decisions the woman is at least partially involved in, coded as 2 = woman alone makes the decision, 1 = woman makes decision with others, 0 = woman does not make the decision. For any decision that may be irrelevant, such as child's health for women without children, we take the average of decisions that remain. This measure was collected beginning in 1997/1998. For kin availability, there are a lot of possible combinations as we follow couples across the waves of data collection. We categorise this variable into seven categories based on original data collection and final follow-up: (1) no kin present (at original data collection nor at final follow-up); (2) wife's parent(s) live in village (both time points); (3) husband's parent(s) live in village (both time points); (4) both sets of parent(s) live in village (both time points); (5) no kin lived in village originally, but kin lived in village by the final follow-up; (6) kin lived in village originally and at follow-up, but which parents had changed; and (7) lived in same village as kin originally, but did not by the final follow-up.

We continue to include the control variables: household wealth (averaged across waves where the couple is followed), wife's education, difference in husband and wife's education, religion, region, wife's age at marriage, number of living children (at first interview), arranged marriage status, urban vs. rural residence and duration of time between original interview and maximum possible follow-up. We also include an indicator for whether the couple had experienced the death of a child during the follow-up period since prior research has found that death of a child significantly increases the likelihood of another birth (DaVanzo et al., 2003) and whether the wife was the one who wanted more offspring initially, as there may be a tendency to have fewer children in low-fertility contexts when conflict exists (Voas, 2003). We exclude woman's age at interview because it is correlated with wife's age at marriage, since women typically enter the dataset once they are married.

## Results

Table 1 presents the descriptive statistics for our two samples; couples who report their fertility intentions (labelled ‘Fertility intentions sample’) and those that are followed to determine the resolution of their differential intentions (labelled ‘Fertility resolutions sample’).

**Table 1.** Descriptive statistics on two samples

		Fertility intentions sample				Fertility resolutions sample		
	Mean (or no.)	SD (or percentage)	Range	Percentage missing	Mean (or no.)	SD (or percentage)	Range	Percentage missing
Desired number of future children (women)	1.25	1.11	0–21	0%	n/a			
Desired number of future children (men)	1.37	1.26	0–29	0%	n/a			
Age difference between partners (husband age − wife age)	4.32	4.12	−11–44	0%	4.29	4.11	−9–30	0%
Women's autonomy (average across waves)	n/a				1.05	0.2	0–1.8	0.1%
Household wealth	0	0.96	−4.7–3.2	2.4%	0.08	0.81	−3.09–2.21	0.3%
Education difference between partners (husband's education level – wife's education level)	0.13	1.19	−4–4	1.6%	0.15	1.25	−4–4	0.8%
Wife's age at marriage	20.58	4.04	0–37	0.7%	20.66	4.03	7–34	<0.1%
Number of currently living children	1.38	1.09	0–8	0.7%	1.36	0.98	0–6	<0.1%
Wife's age at interview	26.1	4.81	11–35	0%	n/a			
Duration of follow-up	n/a				12.51	5.47	7–21	0%
Postmarital residence				1.8%				1.6%
Neolocal	2884	30.4%			525	30.0%		
Matrilocal	3695	39.0%			721	41.2%		
Patrilocal	2902	30.6%			505	28.8%		
Kin availability				11.7%				23.3%
Neither wife's nor husband's parents live in village/at start and end for follow-up	1909	22.40%			224	16.42%		
Wife's parent(s) live in village/at start and end	2363	27.7%			210	15.4%		
Husband's parent(s) live in village/at start and end	2397	28.1%			221	16.2%		
Both wife's and husband's parent(s) live in village/at start and end	1859	21.8%			176	12.9%		
No kin at start, live in same village as kin at end	n/a				58	4.3%		
Live in same village with kin at beginning and change to some new combination of kin	n/a				172	12.6%		
Lived in same village as kin at start, but no kin at end	n/a				303	22.2%		
Wife's education				0.8%				0.6%
None	278	2.9%			57	3.2%		
Grade school	3050	31.8%			640	36.2%		
Junior high	2386	24.9%			419	23.7%		
Secondary school	1756	18.3%			262	14.8%		
Vocational secondary school	1164	12.1%			218	12.3%		
Post-secondary school	947	9.9%			173	9.8%		
Arranged marriage				1.7%				1.4%
Yes	878	9.2%			186	10.6%		
No	8617	90.8%			1568	89.4%		
Urban/rural residence				0%				0%
Rural	4642	47.8%			868	48.8%		
Urban	5012	52.2%			911	51.2%		
Religion				0.1%				0%
Islam	8674	89.9%			1615	90.8%		
Protestant	337	3.5%			67	3.8%		
Catholic	120	1.2%			21	1.2%		
Hindu	485	5.0%			72	4.1%		
Buddhist	25	0.3%			3	0.2%		
Other	6	0.1%			1	<0.1%		
Region				0%				0%
Sumatra	2247	23.3%			439	24.7%		
Java	5232	54.2%			953	53.6%		
Bali and Nusa Tenggara	1186	12.3%			189	10.6%		
Kalimantan	496	5.1%			100	5.6%		
Sulawesi	493	5.1%			98	5.5%		
Wave				0%				
1993	1709	17.7%			n/a			
1997	1014	10.5%						
2000	1657	17.2%						
2007	2676	27.7%						
2014	2598	26.9%						
Child died	n/a							0%
No					1716	96.5%		
Yes					63	3.5%		
Who prefers more offspring	n/a							0%
Wife					781	43.9%		
Husband					998	56.1%		

Fertility intentions sample: sample of couples in first marriage, married monogamously, wife aged 35 or younger, who report number of future offspring desired.

Fertility resolutions sample: sample of couples who are followed owing to divergent fertility preferences where one person can be identified as ‘achieving’ their desired fertility. Couples are followed for at least 7 years, must remain married (to each other), and report their birth information.

SD = standard deviation. n/a = not applicable.

***Question 1: Is there a difference in (a) desired future reproduction and (b) ideal family size among married men and women in Indonesia?***

**Figure 1.**
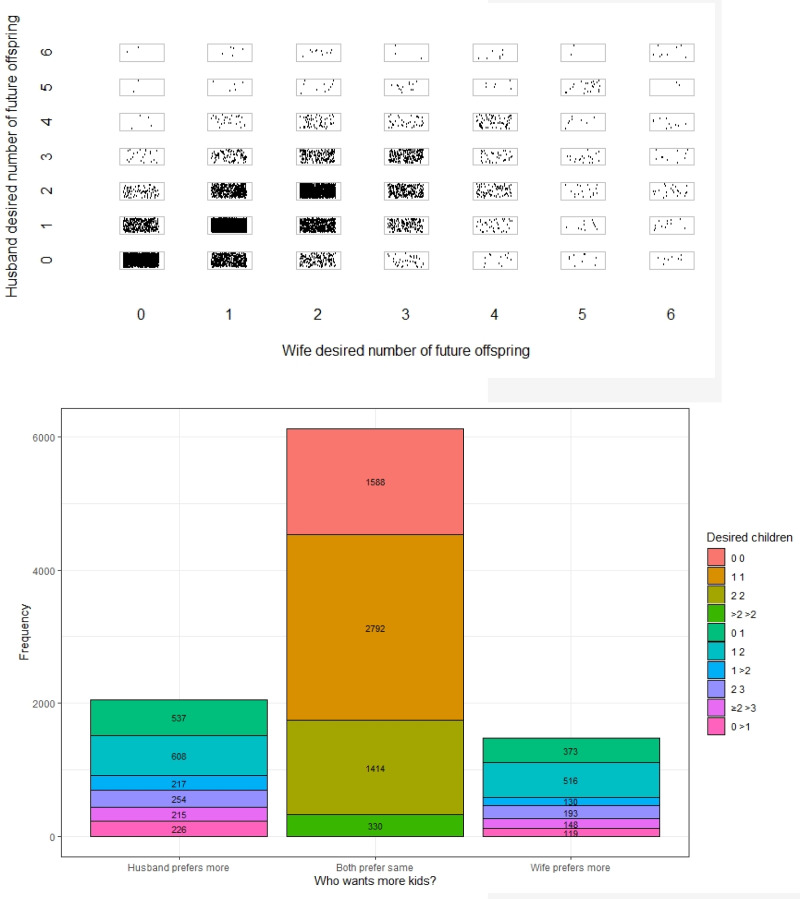
(a) Scatterplot of wife and husband's desired number of future offspring for couples in their first marriage (monogamously married, wife aged 35 years or younger), where each dot represents one couple, using a ‘jitter’ function so that dots overlap minimally. Each box represents a possible outcome, with the most frequent outcome corresponding to both wife and husband desiring one future offspring. We censor the data at six future offspring (all those with preferences above six are grouped into the six category). (b) The frequency of couples that exhibit particular preferences for future children. The legend categories indicate the preference for future children by each partner. For example, ‘0 0’ indicates that both partners want zero future children. The category ‘0 1’ means that one partner wants zero future children while the other wants one more child. The figure then separates by which partner prefers more future children (left and right columns corresponding to *husband prefers more future offspring than his wife* and *wife prefers more future offspring than her husband*, respectively). In the centre column, couples desire the same number of future children – ranging from zero to more than two (‘0 0’ to ‘>2 >2’). Categories with ‘>’ indicate groups. For instance, ‘0 > 1’ refers to all couples where one partner desires zero future offspring and the other partner wants *more than one* future offspring. See Supplementary Materials Table S1 for a frequency distribution of desired future offspring by couples.

Figure 1 presents the preferences for desired future children for couples who fit our sampling criteria (first marriage, monogamous, wife 35 years of age or younger) as a scatter plot (Figure 1a) and a bar chart (Figure 1b). These results show that approximately 64.8% of couples have the same preference for future offspring. An additional 20.7% of couples have husbands who prefer more future offspring than their wives, and 14.5% have wives who prefer more future offspring than their husbands. When differences do occur, 72% of couples exhibit a difference of one child. This suggests that most couples do not report conflict over their future fertility preferences and when different preferences arise, they are typically small (see Supplementary Materials Table S1 for a frequency distribution of fertility preferences). A one-sample test of proportions indicates that, among couples who have differing preferences for future offspring, the proportion of couples where husbands prefer more offspring than wives is statistically greater than 50%, *z*(3823) = 10.75, *p* < 0.001. This remains if we look at each wave separately, where husbands prefer more future offspring than wives in approximately 58.7% of couples where differences occur.

**Figure 2.**
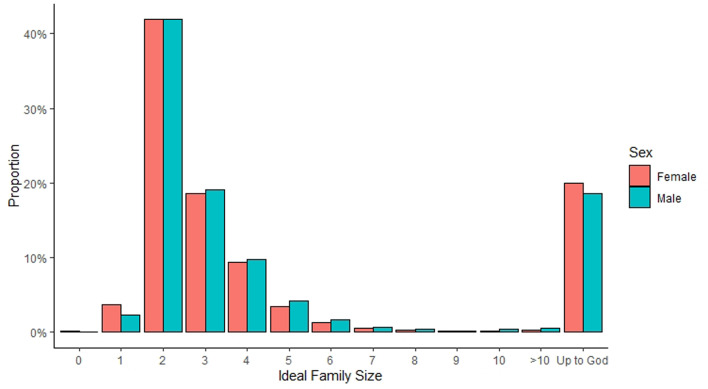
The proportion of responses for ideal family size by men and women. People with an ideal family size of more than 10 children are grouped into ‘>10’. ‘Up to God’ is also a valid response.

We can also examine ideal family size, which will depend less on the number of children couples already have. Figure 2 displays the ideal family size for men and women. This figure shows that women prefer to have one child at slightly higher rates than men, both prefer having two children at the same rate – which is the most frequent response for both men and women – and at higher-order births (three or more), men exhibit the preference slightly more often than woman. Participants could respond that they had no ideal family size and that it is ‘up to God’. We see that women are slightly more likely than men to respond that their ideal family size is ‘up to God’. A *t*-test comparing the averages of men and women who report a numeric value shows that the ideal family size is significantly different. Men (mean, *M* = 3.04, standard deviation, SD = 2.06) report a significantly higher ideal family size than women (*M* = 2.90, SD = 1.67), *t*(33,159) = −6.32, *p* < 0.001. The distribution of ideal family size changes quite substantially across waves (see Supplementary Materials Figure S1), where the number of people who report that it is ‘up to God’ declines from about 33.6% in 1997/1998 to 3.5% in 2014/2015. In contrast, the number of people who report two as the ideal family size goes from 27.2% in 1997/1998 to 55.4% in 2014/2015. There is clearly a shift towards an ideal family size of two.

***Question 2: Who prefers more offspring, and which socioecological variables are associated with differential desired fertility?***

Table 2 presents the results of our multinomial logistic regression model, where our effect sizes represent relative risk ratios (RRR). Our reference category is couples who agree on the number of future offspring. If we first examine our predictions, we see that the wife–husband age difference and postmarital residence are not predictive of conflict over desired fertility. Kin availability is predictive of the wife preferring to have more future children. If she lives in a community with her husband's parent(s), she is significantly more likely to desire greater numbers of offspring than her husband compared with women living in a community without kin (RRR = 1.22, 95% CI [1.00, 1.48]). The effect of having her own parent(s) in the village is quite similar (RRR = 1.20, 95% CI [0.99, 1.46]), where the presence of her own parent(s) is also predictive of her wanting more offspring than her husband. Having both sets of parent(s) around is not associated with conflict over desired fertility. Wife's education is associated with reduced conflict over desired future fertility, where women with secondary school education are less likely to exhibit preferences for more future children than their husbands compared with women with no education (RRR = 0.62, 95% CI [0.40, 0.96]). If we examine our control variables, we see that couples are more likely to agree on the number of future offspring when the couple:(1) practices Hinduism; (2) lives on Java; and (3) has more living children. Couples are less likely to agree on future fertility in 2014/2015, as there is an increased likelihood that men want more offspring than their wives *and* that wives want more offspring than their husbands. If we examine the model predicting the relative risk of the husband preferring more future offspring than the wife, we see that living in an urban area is associated with an increased likelihood that husbands want more children (RRR=1.14, 95% CI [1.00, 1.29]). Since kin availability was not available in the 1997/1998 wave, we report the multinomial logistic model without kin availability in Supplementary Materials Table S2. The results are substantively similar, except that husbands are less likely to desire more future offspring than their wives in 1993/1994 and 1997/1998.

**Table 2.** Multinomial logistic regression model predicting conflict over desired future family size. The reference category is couples who prefer the same number of future children

	Husband prefers more future children			Wife prefers more future children		
	RRR	SE	*p*-Value	RRR	SE	*p*-Value
Husband–wife age difference	0.99	0.01	0.46	1.01	0.01	0.17
Post-marital residence (reference = neolocal)						
Matrilocal	0.99	0.07	0.86	1.00	0.08	0.99
Patrilocal	0.99	0.08	0.92	0.98	0.09	0.82
Kin availability (reference = no parents in village)						
Wife's parent(s) in village	1.08	0.09	0.35	**1**.**20**	**0**.**12**	**0**.**06**
Husband's parent(s) in village	0.95	0.08	0.52	**1**.**22**	**0**.**12**	**0**.**04**
Both sets of parent(s) live in village	0.87	0.08	0.13	0.98	0.11	0.82
Wife's education (reference = no school)						
Grade school	1.20	0.26	0.39	0.77	0.15	0.19
Junior high	1.10	0.24	0.68	**0**.**66**	**0**.**14**	**0**.**05**
Secondary school	1.27	0.29	0.30	**0**.**62**	**0**.**14**	**0**.**03**
Vocational secondary school	1.28	0.31	0.30	**0**.**61**	**0**.**15**	**0**.**04**
Post-secondary	1.42	0.35	0.16	0.80	0.20	0.36
Household wealth	1.03	0.04	0.43	0.99	0.04	0.86
Husband–wife educational difference	1.02	0.03	0.42	1.01	0.03	0.68
Wife's age at marriage	1.00	0.01	0.69	1.01	0.01	0.55
Arranged marriage (reference = not arranged)	1.10	0.12	0.37	1.07	0.13	0.58
Number of living children	0.96	0.04	0.32	**0**.**85**	**0**.**04**	**<0**.**01**
Wife's age (at interview)	1.00	0.01	0.80	1.01	0.01	0.19
Religion (reference = Islam)						
Protestant	1.09	0.17	0.55	1.12	0.19	0.51
Catholic	0.79	0.20	0.34	0.59	0.19	0.11
Hinduism	**0**.**71**	**0**.**11**	**0**.**03**	**0**.**59**	**0**.**11**	**0**.**01**
Buddhism	1.99	0.96	0.15	0.37	0.38	0.34
Other	2.51	3.62	0.52	0.00	0.00	0.99
Region (reference = Sumatra)						
Java	0.90	0.07	0.15	**0**.**73**	**0**.**06**	**<0**.**01**
Bali and Nusa Tenggara	1.17	0.13	0.17	1.00	0.13	0.98
Kalimantan	0.90	0.13	0.46	**0**.**75**	**0**.**12**	**0**.**08**
Sulawesi	1.11	0.16	0.46	0.90	0.15	0.51
Urban (reference = rural)	**1**.**14**	**0**.**07**	**0**.**05**	1.08	0.08	0.30
Wave reported preference (reference = 2000)						
1993	**0**.**80**	**0**.**08**	**0**.**03**	0.88	0.10	0.27
2007	1.14	0.09	0.11	1.08	0.10	0.42
2014	**1**.**45**	**0**.**12**	**<0**.**01**	**1**.**30**	**0**.**13**	**0**.**01**
Constant	**0**.**27**	**0**.**09**	**<0**.**01**	**0**.**24**	**0**.**08**	**<0**.**01**

Note: even though ‘husband prefers more future children’ and ‘wife prefers more future children’ are presented in different columns, they were all included in one multinomial logistic regression model. RRR, Relative risk ratio; SE, standard error; *n* = 7928.

Bold values indicates *p* < 0.10.

***Question 3: How are these differences (when they exist) resolved? Under which socioecological contexts do individuals achieve their preferred number of offspring?***

Of the 1949 couples who disagreed on the number of future offspring (and met our other sampling criteria, see ‘Samples’), there were 1098 (56%) couples where the wife wanted fewer future offspring than her husband and 851 (44%) where the wife wanted more future offspring than her husband. Examining these couples, we see that in about 48% of couples the wife achieved her desired future fertility, while in only 43% of couples did the husband achieve his desired future fertility (see Table 3). The remaining 9% of couples had an intermediate number of offspring between their stated preferences. We can also see that the person who wants fewer offspring is more likely to achieve their preference: in 54% of couples when the wife wants fewer offspring and 53% of couples when the husband wants fewer offspring.

**Table 3.** Of couples that disagree over future fertility, who achieves their desired fertility after at least 7 years?

	W < H	W > H	Total
Wife achieves desired fertility	593 (54%)	335 (39%)	928 (48%)
Compromise	100 (9%)	70 (8%)	170 (9%)
Husband achieves desired fertility	405 (37%)	446 (53%)	851 (43%)
	1098 (100%)	851 (100%)	1949 (100%)

W < H represents couples where the wife desires fewer future offspring than her husband, W > H represents couples where the wife desires more future offspring than her husband. Wife achieves desired fertility includes women who had their desired number of offspring *or* fewer if they are in the W < H category, or the number of women who have their desired number of offspring *or* more if they are in the W > H category. The same criteria hold for ‘husband achieves desired fertility’. Compromise indicates couples where they had a difference in desired fertility of greater than one offspring and had a number in between the wife and husband's desired future fertility.

If we examine the couples where one person ‘achieves their desired fertility’, we can examine whether there are particular traits about a couple that leads the wife or husband to achieve their preference. Table 4 presents the results of our logistic regression model where our outcome is the wife achieved her desired fertility (1) or the husband achieved his desired fertility (0). Given that kin availability is not available for all waves (excluded in 1997/1998), we present the results with kin availability (A) and without (B). The results show that if the wife initially desired more children than her husband, she is significantly less likely to achieve her desired fertility (OR = 0.427, 95% CI [0.34, 0.54]). Counter to predictions, whether the couple lived matrilocally, patrilocally or neolocally does not have a significant effect on whether the wife or husband achieves their desired fertility, nor does the local availability of kin. Similarly, women's autonomy and education do not have significant effects on her ability to achieve her desired number of offspring. The age difference between husbands and wives is significant in Model B, but in the opposite direction than predicted, where a larger difference between husband and wife's age is associated with the wife being more likely to achieve her desired number of offspring (OR = 1.03, 95% CI [1.00, 1.06]). Couples that report their marriage as arranged by others (in Model A) have a reduced likelihood that the wife achieves her preference (OR = 0.67, 95% CI [0.45, 1.00]), which may support hypotheses related to autonomy if arranged marriage is indicative of low autonomy of women. Women who report an older age at marriage are more likely to achieve their desired number of future offspring (OR = 1.03, 95% CI [0.99, 1.07]). We exclude the results from region, religion and urban/rural status (for space), but couples residing in Sulawesi are significantly more likely to be associated with the wife achieving her desired number of offspring. We can also check the sensitivity of these results by examining only couples who have been tracked for 10 or more years. Sample size falls dramatically (by about half), but results show that the effect of the wife being less likely to achieve her desired fertility when her preference for children is higher no longer holds (OR = 1.10, 95% CI [0.79, 1.52]) and the effect of arranged marriage is much reduced (OR = 0.96, 95% CI [0.63, 1.45], see Supplementary Materials Table S3). This suggests that the effect of the person who wants fewer children being able to achieve their desired fertility outcome may be partially explained by not being able to follow couples for a sufficient duration and that arranged marriage effects are not robust. Other effects become less significant, probably owing to reduced statistical power, but their effect sizes remain similar. As wife's age at marriage increases, the odds that she achieves her desired fertility increase (OR = 1.04, 95% CI [0.99, 1.10]) and spousal age difference, where the difference in ages between spouses is greater, is associated with a greater likelihood that the wife achieves her fertility preference (OR = 1.03, 95% CI [1.00, 1.07]).

**Table 4.** Logistic regression model predicted: wife achieves desired fertility (1) vs. husband achieves his desired fertility (0). Couples are included if they were tracked for 7 or more years and if the couple was in their first marriage and remained married. Model A includes the kin availability variable (which is not measured for people who first answered the survey in 1997). Model B excludes this variable and therefore has a larger sample size

	A. Model with kin availability			B. Model without kin availability		
	Odds ratio	SE	*p*-Value	Odds ratio	SE	*p*-Value
Husband–wife age difference	1.02	0.02	0.28	**1**.**03**	**0**.**01**	**0**.**05**
Postmarital residence (reference = neolocal)						
Matrilocal	1.10	0.17	0.51	1.08	0.13	0.54
Patrilocal	0.94	0.15	0.72	0.88	0.12	0.35
Kin availability (reference = no kin at start or end)						
Wife's parent(s) live in village at start and end	1.17	0.26	0.48			
Husband's parent(s) live in village at start and end	1.10	0.24	0.66			
Both sets of parent(s) live in village at start and end	0.88	0.21	0.50			
No kin at start, live in same village as kin at end	0.63	0.20	0.14			
Live with kin at beginning and change to a new combination of kin	1.15	0.27	0.54			
Lived in same village as kin at start, but no kin at end	0.92	0.18	0.68			
Women's autonomy	0.96	0.30	0.90	0.89	0.22	0.64
Household wealth	0.89	0.08	0.19	0.90	0.07	0.20
Wife's education						
Grade school	0.96	0.34	0.92	1.08	0.32	0.80
Junior high	0.99	0.37	0.98	1.03	0.32	0.92
Secondary school	0.95	0.39	0.89	1.11	0.38	0.77
Vocational secondary school	1.05	0.45	0.90	1.17	0.42	0.66
Post-secondary school	1.04	0.48	0.93	1.43	0.55	0.35
Husband–wife educational difference	0.94	0.05	0.30	0.96	0.05	0.38
Wife's age at marriage	**1**.**03**	**0**.**02**	**0**.**09**	**1**.**03**	**0**.**02**	**0**.**07**
Number of living kids at first interview	0.98	0.06	0.76	1.05	0.06	0.37
Arranged marriage (reference = not arranged)	**0**.**67**	**0**.**14**	**0**.**05**	0.84	0.15	0.31
Duration followed up	1.01	0.01	0.58	1.00	0.01	0.99
Child died	1.79	0.59	0.08	1.36	0.37	0.26
Wife desires more offspring	**0**.**43**	**0**.**05**	**<0**.**01**	**0**.**52**	**0**.**05**	**<0**.**01**
Constant	0.65	0.46	0.54	0.57	0.31	0.30
*n*	1318			1724		

Note: models control for region, religion and urban/rural residence.

Bold values indicates *p* < 0.10.

## Discussion

In this paper, we seek to understand the factors that influence conflict over family size and its resolution in Indonesia. A summary of our research questions, hypotheses and results can be found in Table 5. Our findings show that the majority of couples have similar preferences for future offspring, but when differences occur, men are statistically more likely to prefer greater numbers of future offspring. The factors that influence conflict within couples include whether the couple lives in the same village as the wife's or husband's parent(s). Women are more likely to prefer a greater number of future offspring than their husbands when they live in the same village as the wife's or husband's parent(s). The relative risk ratio of 1.2 corresponds to women desiring more future children than their husbands about 14% of the time when they live in a village without either of their parents, but 17% of the time when they live in a community with either the husband's or wife's parents. Increased education for women is associated with a reduced likelihood that women want more children than their husbands, where a baseline rate of 14% of women drops to about 8.7% of women with secondary school education levels wanting more future children than their husbands (after controlling for covariates). We find that few variables significantly predict who will achieve their desired fertility. Overall, the person who wants fewer offspring tends to achieve their preference slightly more often (at least when follow-up is limited to seven years) and wives are slightly more likely to achieve their preference than husbands. Additionally, marrying at a later age or a larger spousal age gap are both associated with an increased likelihood that a woman achieves her desired offspring number.

**Table 5.** Summary of research questions, related hypotheses and results

Research question	Hypothesis	Evidence supports hypothesis?
1. Is there a difference in desired future reproduction and ideal family size among married men and women in Indonesia?	1: Men have higher ideal family sizes than woman, but within reproducing couples, desire a similar number of future offspring	Men have slightly higher ideal family sizes, but also are slightly more likely to have higher desired future fertility within couples
2. Which socioecological variables are associated with differential desired fertility?	2a: Men have lower fertility intentions than women when husbands are significantly older than their wives	No
	2b: Women living patrilocally will have higher desired fertility than their husbands	Yes, when living in a village with husband's kin
	2c: Women living matrilocally will have lower desired fertility than their husbands	No
	2d: Women living near kin will have higher desired fertility than their husbands	Yes
	2e: Women with more education will have lower desired fertility than their husbands	Yes, for women with secondary school level education
3. How are fertility intention differences resolved? Under which socioecological contexts do individuals achieve their preferred number of offspring?	3a: When women have more autonomy, they are more likely to achieve their desired fertility.	No
	3b: When the spousal age gap is larger, men will be more likely to achieve their desired fertility	No
	3c: Women living matrilocally (or near kin) will be more likely to achieve their desired fertility, while women living patrilocally (or away from kin) will be less likely to achieve their desired fertility	No
	3d: In low fertility contexts, couples will be less likely to have more children if they disagree over when to have additional offspring	Yes, but only when restricted to seven years of follow-up

When reflecting on these results in light of our original hypotheses, we find that, on average, men are more likely to prefer greater numbers of future offspring than women, but the majority of couples agree on future reproduction. The result is mirrored in ideal family size, where there are minor differences between men and women, with slightly higher ideal family sizes by 0.14 children on average among men. So, counter to our original hypothesis, we do not find differences between ideal family size and desired future fertility. It is impossible to know how people interpret these questions and whether they think only about children within their current partnership or across multiple partners when answering these questions. Our results do not find differences across these measures, providing suggestive evidence that people perceive these questions in similar ways. Overall, we find small, but significant, differential fertility preferences within a minority of couples.

We also do not find evidence that men have different fertility intentions than woman when they are significantly older, which we predicted based on their decreasing paternity certainty given higher mortality risks. Perhaps our prediction should have focused on different preferences for interbirth interval. Women, who because of their younger age, can expect to live more future years than their much older spouses, may wish to have offspring at longer birth intervals, spacing out births over their reproductive life. Men, who are older and have fewer future years of life, may prefer to have offspring at a faster rate so as to utilise their wives’ reproductive resources before their death. In this case, we might not observe differences in desired future fertility, just differences in ideal birth intervals. Future research should examine whether different interbirth intervals are found for couples with large spousal age gaps.

When we examine predictions based on socioecological contexts, we find evidence that women living near their husband's kin have higher desired fertility than their husbands. This may reflect intergenerational reproductive conflict (Cant & Johnstone, 2008), where younger women who live without kin nearby prefer to increase effort towards direct reproduction. However, our results also show a similar effect of women desiring more offspring when they live in the same village as their parent(s). This is counter to *Hypothesis 2c: Women living matrilocally will have lower desired fertility than their husband* but does conform to *Hypothesis 2d: Women living near kin will have higher desired fertility than their husbands*. This suggests that women desire more offspring than their husbands when they have kin (either their parents or their husband's parents) nearby to help them with allocare or provide financial or emotional support, or are responding to pronatal messaging from kin. An alternative hypothesis is that women who want to have more offspring opt to live near kin. Our results do not support the idea that women are exploited when they live near their husband's kin into having offspring that they do not want to have. Of course, it is possible that women hide their true preferences or report what they view as their inevitable reproductive outcomes.

The results show a significant effect of kin availability, but not postmarital residence. It is possible that postmarital residence, which looks at co-residence immediately following marriage, is a less appropriate measure than local kin availability at the time of interview, given that couples may have already relocated or that kin living nearby may be more indicative of alloparental help, support or pronatal messaging than strictly limiting to co-resident kin. The finding that kin availability of both sets of parents does not significantly predict differential preferences for offspring is surprising given that one would expect if each set of parents by themselves increases the likelihood that women want more children than their husbands, then combined we should see at least similar effects. In reality, we see that, when couples live in communities with both sets of parents, they are slightly more likely to agree on the number of offspring (66% of couples agree, compared with about 62% in other kin categories) and the number of children they want tends to be slightly higher overall. For instance, couples that want more than five additional offspring always live in communities with both sets of parents.

Education, which we predicted would reduce women's desired fertility, is associated with a reduced likelihood that women want more offspring than their husbands. In couples where women have more education, couples are more likely to either agree on future fertility or have the husband desire more future offspring than the wife. This suggests that women's education – a critically important factor in predicting reduced fertility outcomes (Castro Martin, 1995; Colleran & Snopkowski, 2018; Skirbekk, 2008; Snopkowski & Kaplan, 2014) also influences desired fertility, particularly for women.

The resolution of fertility intentions finds few robust predictors. There is no evidence that greater autonomy allows women to achieve their desired fertility, particularly when we examined women's autonomy in household decisions. The factors that were associated with fertility resolutions include age at marriage and spousal age gap, where older women and greater age differences between spouses were associated with women achieving their desired fertility. It is possible that women who marry at older ages are more certain about their reproductive preferences (since they have had more time to think about them) and are unlikely to revise them with time. This also provides evidence against the argument that the fertility transition can be explained as an increase in women's autonomy, whereby women can achieve their lowered desired fertility through greater decision-making power (see also Morgan, Stash, Smith, & Mason, 2002). Contextual factors may influence this finding; Indonesia already had relatively low fertility at the time of the survey (total fertility rate in 1993 was 2.8) and women have long had relatively high levels of autonomy (Stoler, 1977), which may obscure any relationship between autonomy and fertility outcomes.

There is no evidence that living near kin influences a person's ability to achieve their desired fertility. This is counter to some prior interpretations and may mean that this context is unique or that women are generally not exploited into having children they do not want. Finally, we find that couples are more likely to have fewer offspring when couples disagree on the number of children they want to have – but that this result is only robust to following couples for seven years. The effect is eliminated when we follow couples for at least 10 years, suggesting that seven years may not be sufficient time for couples to complete their desired fertility.

As alluded to previously, these results need to be examined in light of the context of Indonesia. Indonesia's fertility was relatively low by 1993/1994 when the first wave of data was collected, which could limit the level of conflict exhibited between couples. Women's autonomy is generally high in this country, allowing women opportunities that may not be available everywhere, which may reduce or eliminate the exploitation of women. Further, polygyny is allowed in Indonesia and may reduce conflict over reproductive decisions (Mulder & Rauch, 2009). This makes it unnecessary for men or their families to try to exploit a woman if they can acquire an additional wife – which may be the easiest route towards higher fertility for men, if it is desired. Contexts, like sub-Saharan Africa, where greater differences in fertility preferences have been identified may be better locations to examine how these preferences are resolved.

There are a variety of limitations to this work. First, people may self-select their partners based on reproductive preference (e.g. McAllister et al., 2012) or their desired future fertility may be the result of prior negotiation and compromise. This means that our analyses cannot pick up prior negotiation and may overemphasise agreement. It is also likely that people revise their preference for offspring over time. These changes in desired fertility may lead to outcomes like ours, where few factors are associated with who achieves their desired outcome. Individual personalities may be more influential in identifying who achieves their preference for offspring (Hutteman, Bleidorn, Penke, & Denissen, 2013). Stochastic processes may play an important role in understanding fertility outcomes (Hruschka & Burger, 2016), which may reduce predictive power in these analyses. Additionally, people may not accurately report their desired fertility, either because they do not contemplate these questions or they try to report preferences that would be deemed socially acceptable, for instance by their community (Kazenin & Kozlov, 2020). While Indonesia is not known for having strong biases for sons or daughters, some research has found that, at least in parts of Indonesia, there is a slight son preference (Guilmoto, 2015). We did not examine how this may have influenced desired fertility or reproductive outcomes, but it would be an important factor to include in countries with strong gender preferences. Finally, we cannot comment on whether differences in fertility preferences are the result of adaptive sexual conflict or the optimisation of factors other than fitness.

In conclusion, there is some evidence of conflict over fertility preferences, but no evidence that conflict is resolved in favour of men over women systematically in Indonesia. The factor that explains differential fertility preferences best is kin availability, suggesting that women's availability of alloparents for support or exposure to pronatal messages influences their strategic preference for more offspring under favourable social conditions.

## Supplementary material

For supplementary material accompanying this paper visit https://doi.org/10.1017/ehs.2021.27. click here to view supplementary material

## Research transparency and reproducibility

The data for the Indonesia Family Life Survey is available at: https://www.rand.org/well-being/social-and-behavioral-policy/data/FLS/IFLS/access.html
