# Supplementary material for: Fertility intentions and outcomes in Indonesia: Evolutionary perspectives on sexual conflict
Source: Evol Hum Sci. 2021 May 6;3:e33. doi: 10.1017/ehs.2021.27 (PMC10427277; doi:10.1017/ehs.2021.27)
Supplement: Supplementary file 1 [file S2513843X2100027Xsup001.docx]

**Supplementary Materials**

Table S1: Frequency distribution of couples (in their first marriage, married monogamously) that exhibit particular preferences for future children. Preferences can be read as the first number indicates the preference for one partner and the second number is the preference for the second partner (when their preferences differ).

| **Desired Kids** | | |
| --- | --- | --- |
| Preferences for (future) children – couple has same preference | Frequency | |
| Both 0 | 2325 | |
| Both 1 | 2894 | |
| Both 2 | 1476 | |
| Both 3 | 243 | |
| Both 4 | 71 | |
| Both 5 | 23 | |
| Both 6 | 2 | |
| Both 9 | 1 | |
| Preferences for (future) children | Husband prefers more | Wife prefers more |
| 0, 1 | 628 | 414 |
| 0, 2 | 205 | 104 |
| 0, 3 | 46 | 25 |
| 0, >3 | 34 | 10 |
| 1, 2 | 625 | 538 |
| 1, 3 | 160 | 100 |
| 1, >3 | 62 | 36 |
| 2, 3 | 259 | 197 |
| 2, 4 | 86 | 62 |
| 2, >4 | 44 | 20 |
| 3, 4 | 39 | 40 |
| 3, >4 | 31 | 16 |
| 4, 5 | 9 | 7 |
| 4, >5 | 10 | 7 |
| >4, >6 | 6 | 3 |

> indicates that the preference is greater than this value. This groups couples with low frequency and high future fertility preferences.


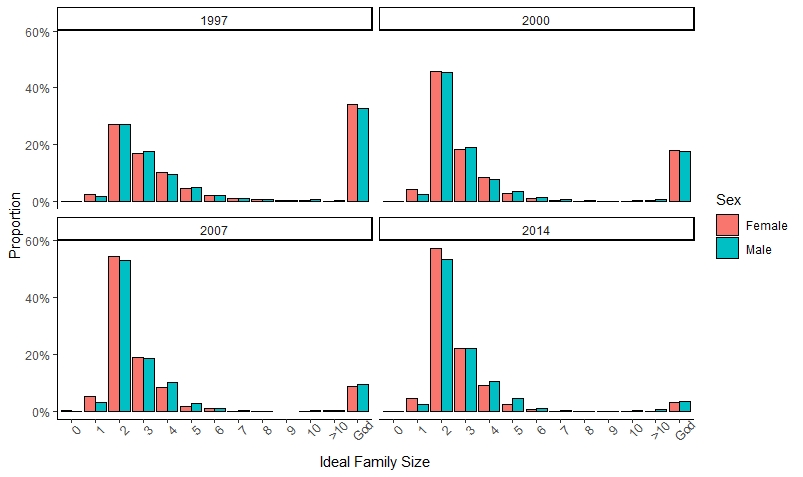


Figure S1: The proportion of responses for ideal family size by men and women across waves. People with an ideal family size of more than 10 children are grouped into “>10”. “Up to God” is indicated by “God”. People are only included in the wave in which they first answered the question about their ideal family size. This question was not included in the 1993/1994 wave of data collection.

Table S2: Multinomial logistic regression model predicting conflict over desired future family size without kin availability variable (since it was excluded in 1997/1998 wave). Reference category = couples who prefer the same number of future children. Note: even though ‘husband prefers more future children’ and ‘wife prefers more future children’ are presented in different columns, they were all included in one multinomial logistic regression model. RRR = relative risk ratio, SE = standard error, n=9023

|  | Husband prefers more future children | | | Wife prefers more future children | | |
| --- | --- | --- | --- | --- | --- | --- |
|  | RRR | SE | p-value | RRR | SE | p-value |
| Wife-Husband Age Difference | 0.992 | 0.007 | 0.288 | 1.009 | 0.008 | 0.255 |
| Post-marital residence (ref = Neolocal) | | | |  |  |  |
| Matrilocal | 1.000 | 0.066 | 0.994 | 0.981 | 0.074 | 0.795 |
| Patrilocal | 0.962 | 0.067 | 0.577 | 0.982 | 0.077 | 0.822 |
| Wife's education (ref = no school) | | | |  |  |  |
| Grade School | 1.221 | 0.240 | 0.308 | 0.860 | 0.162 | 0.422 |
| Jr High | 1.136 | 0.233 | 0.536 | 0.752 | 0.151 | 0.155 |
| Secondary school | 1.308 | 0.278 | 0.206 | 0.703 | 0.149 | 0.096 |
| Vocational secondary school | 1.298 | 0.289 | 0.240 | 0.698 | 0.157 | 0.110 |
| Post-secondary | 1.401 | 0.323 | 0.144 | 0.869 | 0.203 | 0.549 |
| Household wealth | 1.040 | 0.037 | 0.261 | 1.005 | 0.039 | 0.906 |
| Wife-Husband Educational Difference | 1.011 | 0.026 | 0.678 | 1.019 | 0.030 | 0.524 |
| Wife's age at first marriage | 1.006 | 0.011 | 0.614 | 1.009 | 0.012 | 0.482 |
| Arranged marriage (ref = not arranged) | 1.094 | 0.110 | 0.374 | 1.131 | 0.126 | 0.267 |
| Number of living children | 0.942 | 0.036 | 0.117 | **0.827** | **0.036** | **<0.001** |
| Wife's age (at interview) | 0.998 | 0.009 | 0.810 | 1.016 | 0.011 | 0.128 |
| Religion (ref = Islam) | | |  |  |  |  |
| Protestant | 1.113 | 0.162 | 0.461 | 1.141 | 0.184 | 0.415 |
| Catholic | 0.903 | 0.212 | 0.664 | 0.561 | 0.183 | 0.077 |
| Hinduism | **0.666** | **0.102** | **0.008** | **0.580** | **0.103** | **0.002** |
| Buddhism | 1.602 | 0.742 | 0.309 | 0.600 | 0.454 | 0.499 |
| Other | 2.296 | 2.159 | 0.377 | 0.000 | 0.002 | 0.984 |
| Region (ref = Sumatra) | | |  |  |  |  |
| Java | 0.883 | 0.062 | 0.076 | **0.729** | **0.057** | **<0.001** |
| Bali & Nusa Tenggara | 1.114 | 0.120 | 0.316 | 1.010 | 0.121 | 0.932 |
| Kalimantan | 0.877 | 0.118 | 0.327 | 0.811 | 0.120 | 0.158 |
| Sulawesi | 1.090 | 0.148 | 0.529 | 0.930 | 0.144 | 0.639 |
| Urban (ref=rural) | **1.152** | **0.069** | **0.018** | 1.097 | 0.075 | 0.174 |
| wave reported (ref = 2000) | | |  |  |  |  |
| 1993/1994 | **0.806** | **0.080** | **0.030** | 0.887 | 0.099 | 0.279 |
| 1997/1998 | **0.735** | **0.081** | **0.005** | 0.868 | 0.105 | 0.242 |
| 2007/2008 | 1.120 | 0.091 | 0.164 | 1.073 | 0.100 | 0.451 |
| 2014/2015 | **1.436** | **0.120** | **<0.001** | **1.301** | **0.125** | **0.006** |
| Constant | **0.267** | **0.080** | **<0.001** | **0.232** | **0.073** | **<0.001** |

Table S3: Logistic regression model predicting whether the wife achieves her desired number of future offspring (1) vs. the husband achieves his desired number of future offspring (0) for couples that could be examined over 10 years (or more)

|  | Model with kin availability | | | Model without kin availability | | |
| --- | --- | --- | --- | --- | --- | --- |
|  | OR | SE | p-value | OR | SE | p-value |
| Husband-Wife Age Difference | 1.022 | 0.024 | 0.348 | **1.032** | **0.018** | **0.078** |
| Postmarital residence (ref = Neolocal) | | | |  |  |  |
| Matrilocal | 0.868 | 0.188 | 0.515 | 0.898 | 0.148 | 0.516 |
| Patrilocal | 0.734 | 0.174 | 0.191 | 0.767 | 0.139 | 0.143 |
| Kin Availability (ref = no kin at start or end) | | | |  |  |  |
| Wife's parent(s) live in village at start & end | 1.362 | 0.467 | 0.367 |  |  |  |
| Husband's parent(s) live in village at start & end | 1.342 | 0.460 | 0.390 |  |  |  |
| Both sets of parent(s) live in village at start & end | 1.071 | 0.405 | 0.856 |  |  |  |
| No kin at start, live in same village as kin at end | 0.713 | 0.305 | 0.428 |  |  |  |
| Live in same village with kin at beginning and change to some new combination of kin | 1.289 | 0.407 | 0.422 |  |  |  |
| Lived in same village as kin at start, but no kin at end | 0.946 | 0.247 | 0.832 |  |  |  |
| Women's autonomy | 1.135 | 0.540 | 0.790 | 0.841 | 0.281 | 0.603 |
| Household wealth | 0.843 | 0.127 | 0.256 | 0.902 | 0.103 | 0.367 |
| Wife's Education | |  |  |  |  |  |
| Grade School | 0.823 | 0.336 | 0.633 | 1.031 | 0.347 | 0.927 |
| Jr High | 0.750 | 0.333 | 0.517 | 0.868 | 0.318 | 0.700 |
| Secondary School | 0.588 | 0.305 | 0.306 | 0.876 | 0.360 | 0.746 |
| Vocational Secondary School | 0.903 | 0.492 | 0.852 | 1.024 | 0.448 | 0.957 |
| Post-secondary School | 1.108 | 0.701 | 0.871 | 1.937 | 0.990 | 0.196 |
| Husband -Wife Educational Difference | 0.971 | 0.081 | 0.724 | 0.958 | 0.060 | 0.499 |
| Religion (ref = Islam) | |  |  |  |  |  |
| Protestant | 0.894 | 0.414 | 0.808 | 1.038 | 0.389 | 0.920 |
| Catholic | 4.810 | 5.426 | 0.164 | 3.379 | 2.762 | 0.136 |
| Hinduism | 1.338 | 0.668 | 0.560 | 1.412 | 0.598 | 0.415 |
| Buddhism | 1.351 | 1.964 | 0.836 | 1.075 | 1.540 | 0.960 |
| Region (ref = Sumatra) | |  |  |  |  |  |
| Java | 1.179 | 0.249 | 0.436 | 1.227 | 0.214 | 0.242 |
| Bali & Nusa Tenggara | 1.215 | 0.440 | 0.590 | 1.592 | 0.460 | 0.108 |
| Kalimantan | 0.610 | 0.244 | 0.217 | 0.793 | 0.257 | 0.474 |
| Sulawesi | 1.466 | 0.618 | 0.364 | 1.285 | 0.432 | 0.456 |
| Wife's age at marriage | 1.045 | 0.030 | 0.126 | 1.025 | 0.023 | 0.252 |
| Number of living kids at first interview | 1.015 | 0.089 | 0.869 | 1.079 | 0.075 | 0.271 |
| Arranged marriage (ref = not arranged) | 0.740 | 0.193 | 0.248 | 0.959 | 0.202 | 0.843 |
| Urban (ref = rural) | 1.200 | 0.235 | 0.350 | 1.130 | 0.168 | 0.413 |
| Duration followed up | 1.024 | 0.029 | 0.393 | 1.010 | 0.022 | 0.655 |
| Child died | 1.482 | 0.630 | 0.355 | 1.087 | 0.357 | 0.799 |
| Wife desires more offspring | 1.098 | 0.184 | 0.576 | 1.069 | 0.142 | 0.616 |
| Constant | 0.235 | 0.253 | 0.179 | 0.430 | 0.344 | 0.292 |
| n | 630 | | | 969 | | |
